# Mouse Anaphylactic Hypotension Is Characterized by Initial Baroreflex Independent Renal Sympathoinhibition Followed by Sustained Renal Sympathoexcitation

**DOI:** 10.3389/fphys.2017.00669

**Published:** 2017-09-07

**Authors:** Tao Zhang, Mamoru Tanida, Kunitoshi Uchida, Yoshiro Suzuki, Wei Yang, Yuhichi Kuda, Yasutaka Kurata, Makoto Tominaga, Toshishige Shibamoto

**Affiliations:** ^1^Department of Physiology II, Kanazawa Medical University Uchinada, Japan; ^2^Department of Colorectal and Hernia Surgery, The Fourth Affiliated Hospital of China Medical University Shenyang, China; ^3^Division of Cell Signaling, Okazaki Institute for Integrative Bioscience, National Institute for Physiological Sciences, National Institutes of Natural Sciences Okazaki, Japan; ^4^Department of Physiological Science and Molecular Biology, Fukuoka Dental College Fukuoka, Japan; ^5^Department of Infectious Disease, The Sheng Jing Hospital of China Medical University Shenyang, China

**Keywords:** autonomic nerves, baroreflex, mice, vagal afferents, TRPV1, anaphylaxis

## Abstract

**Aim:** The hemodynamic response to mouse systemic anaphylaxis is characterized by an initial hypertension followed by sustained hypotension. However, the defense mechanisms of the sympathetic nervous system against this circulatory disturbance is not known. Here, we investigated the renal sympathetic nerve activity (RSNA) response to mouse systemic anaphylaxis, along with the roles of carotid sinus baroreceptor, vagal nerves and the transient receptor potential vanilloid type 1 channel (TRPV1).

**Methods:** Male ovalbumin-sensitized C57BL/6N mice were used under pentobarbital anesthesia. RSNA, systemic arterial pressure (SAP) and heart rate (HR) were continuously measured for 60 min after the antigen injection.

**Results:** Within 3 min after antigen injection, RSNA decreased along with a transient increase in SAP. Thereafter, RSNA showed a progressive increase during sustained hypotension. In contrast, HR continuously increased. Sinoaortic denervation, but not vagotomy, significantly attenuated the renal sympathoexcitation and tachycardia from 30 and 46 min, respectively, after antigen. The responses of RSNA, SAP and HR to anaphylaxis were not affected by pretreatment with a TRPV1 inhibitor, capsazepine, or by genetic knockout of TRPV1.

**Conclusion:** The mouse systemic anaphylaxis causes a biphasic RSNA response with an initial baroreflex-independent decrease and secondary increase. The antigen-induced sympathoexcitation and tachycardia at the late stage are partly mediated by carotid sinus baroreceptors. Either vagal nerve or TRPV1 does not play any significant roles in the RSNA and HR responses in anesthetized mice.

## Introduction

Anaphylactic shock is a life-threatening allergic cardiovascular disorder developing severe and prolonged hypotension (Brown, 1995). The sympathetic nervous system plays an important role in blood pressure recovery during anaphylactic hypotension, as supported by animal studies: chemical sympathectomy and α1-adrenoceptor antagonists deteriorate anaphylactic hypotension in anesthetized rats (Wang et al., 2013). Indeed, during anaphylactic hypotension, the renal and lumbar sympathetic nerve activity increases in rats (Sun et al., 2014; Song et al., 2016) and plasma catecholamine levels elevate in rats and pigs (Jacobsen et al., 1995; Zhang W. et al., 2011). In contrast, renal sympathetic nerve activity (RSNA) does not increase in response to anaphylactic hypotension in anesthetized dogs (Koyama et al., 1990). Therefore, there are species differences in the sympathetic response to anaphylaxis. However, it is not known how RSNA responds to anaphylactic hypotension in mice, which are currently most frequently used for the gene-based study of systemic anaphylaxis (Cauwels et al., 2006; Cui et al., 2013). On the other hand, we previously reported that the hemodynamic characteristics of mouse systemic anaphylaxis are the initial short-lasting hypertension followed by sustained hypotension (Liu et al., 2007; Wang et al., 2014). The initial hypertension is caused by an increase in cardiac output, but not systemic vasoconstriction, without significant changes in total peripheral resistance, while the subsequent hypotension is ascribed to a decrease in cardiac output, which was accompanied by an increase in the total peripheral resistance (Wang et al., 2014). Based on these findings, we here hypothesized that RSNA, which regulates the tonus of the renal artery, the representative resistance artery, shows the baroreflex-dependent biphasic response of the initial decrease in response to the initial hypertension, and the subsequent increase, which responds to the sustained hypotension so as to increase the total peripheral resistance.

The afferent pathways of the carotid sinus nerves and vagal nerves seem to be involved in modulation of systemic anaphylaxis (Castex et al., 1995; Potas et al., 2004; Sun et al., 2014). The vagal afferents participate in regulation of intestinal motility during anaphylaxis in anesthetized rats (Castex et al., 1995). The arterial baroreceptors contribute to the increase of RSNA during anaphylactic hypotension in rats (Potas et al., 2004; Sun et al., 2014). However, it remains unknown whether the carotid sinus nerves or vagal nerves are involved in the regulation of the efferent sympathetic nerve activity during anaphylactic hypotension in anesthetized mice.

Recently, there is increasing evidence that many of the inflammatory mediators released in anaphylactic reactions involve the transient receptor potential vanilloid type 1 channel (TRPV1) (Smith and Nilius, 2013), expressed on not only afferent sensory nerves (Numazaki and Tominaga, 2004), but also baroreceptor reflex pathways such as the arterial baroreceptor afferent (Sun et al., 2009) and vagal afferent (Zhang et al., 2004; Hermes et al., 2016). TRPV1 is stimulated by heat, low pH and many compounds (Caterina et al., 1997; Tominaga et al., 1998). Recently, anaphylactic mediators such as histamine (Kim et al., 2004; Shim et al., 2007), serotonin (Salzer et al., 2016), platelet-activating factor (Marotta et al., 2009), and prostaglandins (Moriyama et al., 2005) sensitize and activate TRPV1. On the other hand, TRPV1 is reported to be involved in regulations of hemorrhagic shock via the baroreceptors (Akabori et al., 2007) and vagal afferents (Zhang et al., 2017). Therefore, these lines of evidence suggest that afferent TRPV1 participates in modulation of anaphylactic hypotension. Little is known, however, about roles of TRPV1 in the sympathetic regulation of anaphylactic hypotension.

In the present study, we firstly clarified the RSNA response to anaphylactic hypotension in anesthetized mice. Second, we determined the possible regulatory roles of baroreflex and TRPV1 in this response by surgical, pharmacologic and genetic approaches.

## Materials and methods

### Animals

Male C57BL/6N mice (body weight, 23 ± 2 g; *n* = 66), as well as male TRPV1 knockout (TRPV1^−/−^) mice (body weight, 20 ± 2 g; *n* = 6) backcrossed in C57BL/6N mice, were used in this study. TRPV1^−/−^ mice were originally given by Dr. D. Julius (University of California, San Francisco). Mice were maintained at 23°C under pathogen-free conditions on a 12:12-h dark/light cycle and allowed food and water *ad libitum*. The experiments conducted in the present study were approved by the Animal Research Committee of Kanazawa Medical University (2016-17).

### Sensitization

Mice were actively sensitized by the subcutaneous injection of an emulsion made by mixing aluminum potassium sulfate adjuvant (2 mg) with 0.01 mg ovalbumin (grade V; Sigma Chemical Co., St. Louis, MO, USA) dissolved in physiological saline (0.2 ml) (Wang et al., 2014). The antigen emulsion was injected again 1 week after the first antigen injection. Non-sensitized mice were injected with aluminum potassium sulfate adjuvant and ovalbumin-free saline. One week after the second injection, the mice were used for the following experiments.

### Surgical preparation

Mice were anesthetized with pentobarbital sodium (60 mg/kg, i.p.); the adequacy of the level of anesthesia was ensured by the lack of withdrawal reflexes to tail pinch. The body temperature was maintained at 36–37°C using a heating pad and was monitored using a thermometer inserted into the rectum. The trachea was cannulated and animals were allowed to breathe oxygen-enriched air. Polyethylene catheters were inserted into the right external jugular vein and the right femoral artery for a continuous infusion of saline (40 ml/kg/h) or injections of drugs and for measurement of the systemic arterial pressure (SAP), respectively.

RSNA was measured as previously reported (Tanida et al., 2013). In brief, the left renal nerve was exposed retroperitoneally through a left flank incision using a dissecting microscope. The nerve was cut and the proximal end was attached to a pair of stainless steel wire electrodes and then connected to the electrodes. The recording electrodes and the nerve were fixed with a silicone gel (liquid A & liquid B, Kagawa kikai Co. JAPAN) to prevent drying and for electrical insulation. The electrical change in the renal nerve was amplified 50,000–100,000 times with a bandpass of 100–1,000 kHz and was monitored by an oscilloscope. The raw data of the nerve activity were converted to standard pulses by a window discriminator. Both the discharge rate and the neurogram were sampled with a Power-Lab analog-to-digital converter for data recording and data analysis on a computer. The nerve activity was rectified, integrated, and normalized to be shown as a percentage of the baseline nerve activity. To ensure that the post-ganglionic efferent sympathetic nerve activity was recorded, hexamethonium chloride (30 mg/kg) was injected intravenously, and the background noise, which was determined 30–60 min after the animal was euthanized, was subtracted. SAP and heart rate (HR) were also sampled using the Power-Lab and were stored on a hard disk for off-line analysis.

### Sinoaortic denervation (SAD) and vagotomy

After the neurovascular trunk in the neck was exposed, bilateral SAD was performed by cutting the carotid sinus nerves, the aortic depressor nerves and the recurrent laryngeal nerves, and by stripping the arterial walls in the carotid sinus region and painting them with 10% phenol. For vagotomy, the cervical vagi were sectioned bilaterally. For intact mice, the neck was exposed without sectioning the nerves. Successful SAD was confirmed by the absence of significant reflex changes in HR and RSNA in response to SAP changes induced by intravenous injections of sodium nitroprusside (SNP, 60 μg/kg) and phenylephrine (PNE, 0.8 μg/kg). Mice in which effective SAD was not confirmed were excluded from the analysis.

### Experimental protocol

After surgery, the baseline was measured for at least 15 min prior to the intravenous injection of the ovalbumin antigen (dose, 0.1 mg/ 50 μl saline; injection time, 5 s). The SAP, mean arterial pressure (MAP), RSNA and HR were continuously measured for 60 min after antigen injection.

The sensitized mice were assigned to the following groups: (1) anaphylaxis, (2) intact, (3) SAD, (4) vagotomy, (5) wild type, and (6) TRPV1^−/−^. The non-sensitized mice were assigned to the control group. In the pharmacological investigation (Akabori et al., 2007; Wang et al., 2008), the vehicle (saline containing 10% Tween 80/10% ethanol) and the TRPV1 antagonist capsazepine (CPZ; dissolved in the vehicle, 9 mg/kg) was intravenously administered at 2 min before an intravenous injection of the antigen into the sensitized mice for the vehicle anaphylaxis and CPZ anaphylaxis group, respectively. For the vehicle and CPZ control studies, the vehicle or CPZ was injected into the non-sensitized mice at 2 min before antigen injection.

In order to depict the characteristics of mouse systemic anaphylaxis clearly, we compared the RSNA response to antigen with that when MAP was similarly manipulated. To mimic the antigen-induced changes in MAP, the initial hypertension was induced by an initial intravenous injection of PNE, while the subsequent hypotension by bleeding through the right femoral artery catheter with a syringe pre-rinsed with heparin at an appropriate speed in the intact and SAD mice.

### Statistical analysis

Results are expressed as means ± SD. Percent changes from baseline values were calculated for RSNA. For the analysis of the variables after the antigen injection, intragroup comparison was performed by one-way analysis of variance and a *P*-value less than 0.05 was considered significant. When a significant difference was obtained, Fisher post hoc test was performed. Between-group comparison was performed by two-way analysis of variance for repeated measures followed by the Bonferroni *post hoc* test.

## Results

### RSNA and cardiovascular responses to anaphylaxis in mice

**Figure 1:**
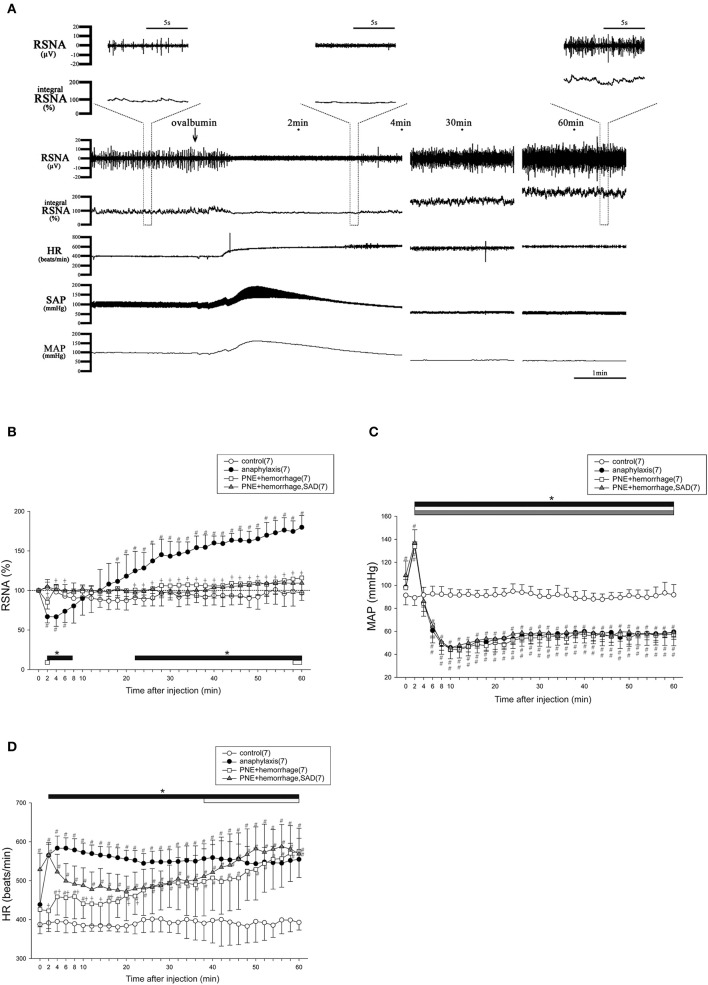
The changes in RSNA and hemodynamic variables after antigen injection in anesthetized mice. The typical raw data of RSNA and cardiovascular responses to anaphylaxis are presented, and expanded raw data of RSNA are represented in upper area **(A)**. Time-course data of the changes in RSNA **(B)**, MAP **(C)**, and HR **(D)** after antigen injection or treatment of PNE injection and hemorrhage in mice are also shown. ●, the sensitized mice (anaphylaxis); ○, the nonsensitized mice (control); □, mice treated with PNE injection and hemorrhage (PNE + hemorrhage); ▲, SAD mice treated with PNE injection and hemorrhage (PNE + hemorrhage, SAD). The numbers of mice used are given in parentheses. Values are expressed as means ± SD. ^#^*P* < 0.05 vs. the nonsensitized mice (control). ^†^*P* < 0.05 the anaphylaxis group vs. the PNE + hemorrhage group. ^*^P < 0.05 vs. the baseline; the black bar, white bar and gray bar show the significant changes in anaphylaxis group, PNE + hemorrhage group, and PNE + hemorrhage + SAD group, respectively.

Figure 1A shows representative recordings of the changes in the variables measured during the first 4, 29–31, and 59–61 min after an antigen challenge in a sensitized mouse. After antigen injection, RSNA showed an initial decrease and a secondary increase. On the contrary, SAP initially increased and then decreased. In contrast, HR sustainably increased after antigen challenge (Figure 1A). Figures 1B–D show the time-course changes in RSNA, MAP and HR, respectively, for the anaphylaxis mice and non-sensitized control mice for 60 min after antigen injection. In the anaphylaxis group, the RSNA began to decrease to 66% of the baseline within 2 min after antigen injection, and then recovered to the baseline around 10 min, followed by a progressive increase reaching 180% of the baseline at 60 min (Figure 1B). In contrast, the MAP initially increased by 37 ± 19 mmHg from the baseline values of 98 ± 8 mmHg at 2 min after antigen injection and then decreased to the nadir level of 45 ± 10 mmHg at 10 min (Figure 1C) with a little recovery at the end of the experimental period. The HR increased rapidly after antigen injection and remained elevated throughout the experimental period (Figure 1D). In the non-sensitized control mice, antigen injection did not evoke any significant changes in the RSNA, MAP, or HR. In the sensitized mice, an injection of the vehicle (saline) alone also did not affect these parameters (RSNA, 101 ± 12% at 60 min after injection; MAP, 86 ± 16 mmHg at baseline and 80 ± 6 mmHg at 60 min after injection; HR, 438 ± 26 beats/min at baseline and 458 ± 23 beats/min at 60 min after injection).

To mimic the biphasic MAP response to the antigen, an intravenous injection of PNE followed by hemorrhage were performed in the intact and SAD mice. Figures 1B–D shows the time-course changes in the variables for the intact and SAD groups for 60 min after initial PNE injection and later hemorrhage. In both groups, a biphasic MAP response was evoked in the same way as that of the anaphylaxis group (Figure 1C). The RSNA in the intact mice showed the biphasic response with an initial decrease followed by a secondary increase. However, this secondary increase in RSNA was significantly smaller than that in the anaphylaxis group (Figure 1B). Moreover, the initial decrease in the RSNA was abolished in the SAD mice (Figure 1B). The HR in the intact mice increased at the late stage of hemorrhage, while HR in the SAD mice did not change throughout the experimental period (Figure 1D).

With subcutaneous injections of antigen in sensitized mice, 0.1 mg antigen (100 μl), the same dose as in the intravenous injection study, did not affect the RSNA, MAP, or HR, whereas 10 mg antigen (100 μl) caused the responses similar to those induced by the intravenous injection of antigen with the biphasic changes in the RSNA and MAP, and a progressive increase in HR (Supplemental Figure 1).

### Effects of SAD or vagotomy on renal sympathetic and cardiovascular responses to anaphylaxis in mice

**Figure 2:**
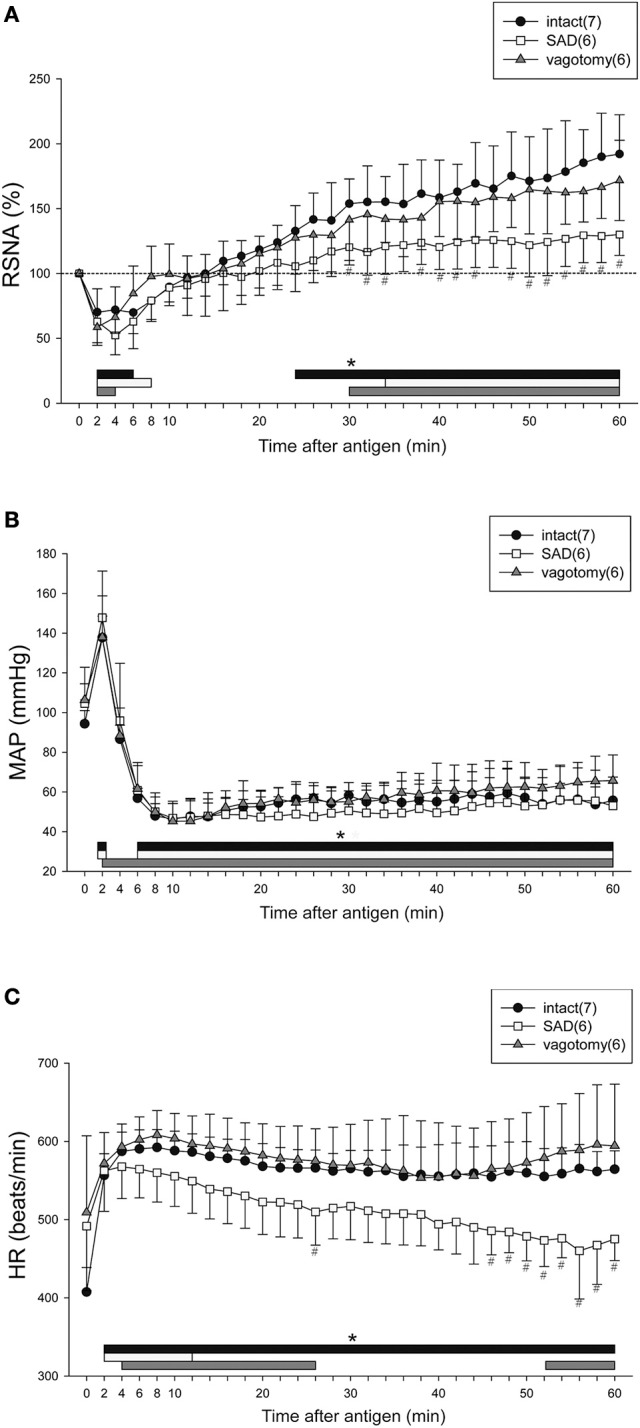
Renal sympathetic and cardiovascular responses to anaphylactic hypotension in the intact, vagotomized, and SAD mice. Summary of the changes in RSNA **(A)**, MAP **(B)**, and HR **(C)** after antigen injection in the intact mice (●), vagotomized mice (▲), and SAD mice (□). The numbers of mice used are given in parentheses. Values are expressed as means ± SD. ^#^*P* < 0.05 vs. the intact mice. ^*^P < 0.05 vs. the baseline; the black bar, white bar and gray bar show the significant changes in intact mice, SAD mice and vagotomized mice, respectively.

Figure 2 shows the time-course changes in the variables for the intact, SAD and vagotomy groups for 60 min after antigen injection. In all sensitized mice with or without intact neuraxis, the RSNA showed the biphasic response to the antigen with an initial decrease followed by a secondary increase (Figure 2A). Although there was no difference in the initial decrease of RSNA between SAD and intact mice, the secondary increase of RSNA in the SAD mice during the period of 30–60 min after antigen injection was significantly smaller than that in the intact mice (Figure 2A). In vagotomized mice, the RSNA response was similar to that of the intact mice throughout the experimental period. In all these three groups, the antigen injection evoked a biphasic MAP response with an initial increase followed by a gradual decrease to the nadir level about 10 min after antigen injection (Figure 2B).

After antigen injection, HR increased in all three groups, but significant differences in HR were found at the late phase of anaphylaxis (46–60 min) between the intact mice and SAD mice (Figure 2C). Taken together, SAD attenuated the responses of both RSNA and HR to anaphylactic hypotension.

With respect to the baroreflex induced by an intravenous injection of SNP or PNE in each group, SNP-induced hypotension caused increases in RSNA and HR in intact mice and vagotomized mice (intact mice; peak response of RSNA 163 ± 23%, peak response of MAP −50 ± 6 mmHg, peak response of HR 34 ± 18 beats/min, ΔRSNA/ΔMAP = −1.3 ± 0.6, ΔHR/ΔMAP = −0.7 ± 0.5, vagotomized mice; peak response of RSNA 165 ± 33%, peak response of MAP −59 ± 13 mmHg, peak response of HR 21 ± 12 beats/min, ΔRSNA/ΔMAP = −1.2 ± 0.8, ΔHR/ΔMAP = −0.4 ± 0.2), while these responses were abolished by SAD (peak response of RSNA 97 ± 4%, peak response of MAP −59 ± 9 mmHg, peak response of HR 2 ± 3 beats/min, ΔRSNA/ΔMAP = 0.04 ± 0.07, ΔHR/ΔMAP = −0.05 ± 0.05). In addition, sympathoinhibitory and bradycardia responses to SAP elevation evoked by an intravenous injection of PNE were observed in the intact mice and vagotomized mice (intact mice; peak response of RSNA 63 ± 9%, peak response of MAP 42 ± 5 mmHg, peak response of HR −26 ± 11 beats/min, ΔRSNA/ΔMAP = −0.89 ± 0.27, ΔHR/ΔMAP = −0.64 ± 0.24, vagotomized mice; peak response of RSNA 63 ± 13%, peak response of MAP 44 ± 6 mmHg, peak response of HR −21 ± 11 beats/min, ΔRSNA/ΔMAP = −0.85 ± 0.38, ΔHR/ΔMAP = −0.5 ± 0.24), but not in the SAD-treated mice (peak response of RSNA 96 ± 4%, peak response of MAP 44 ± 7 mmHg, peak response of HR 6 ± 8 beats/min, ΔRSNA/ΔMAP = −0.07 ± 0.1, ΔHR/ΔMAP = −0.15 ± 0.08).

### Effects of a TRPV1 receptor antagonist, CPZ, on renal sympathetic and cardiovascular responses to anaphylaxis in mice

**Figure 3:**
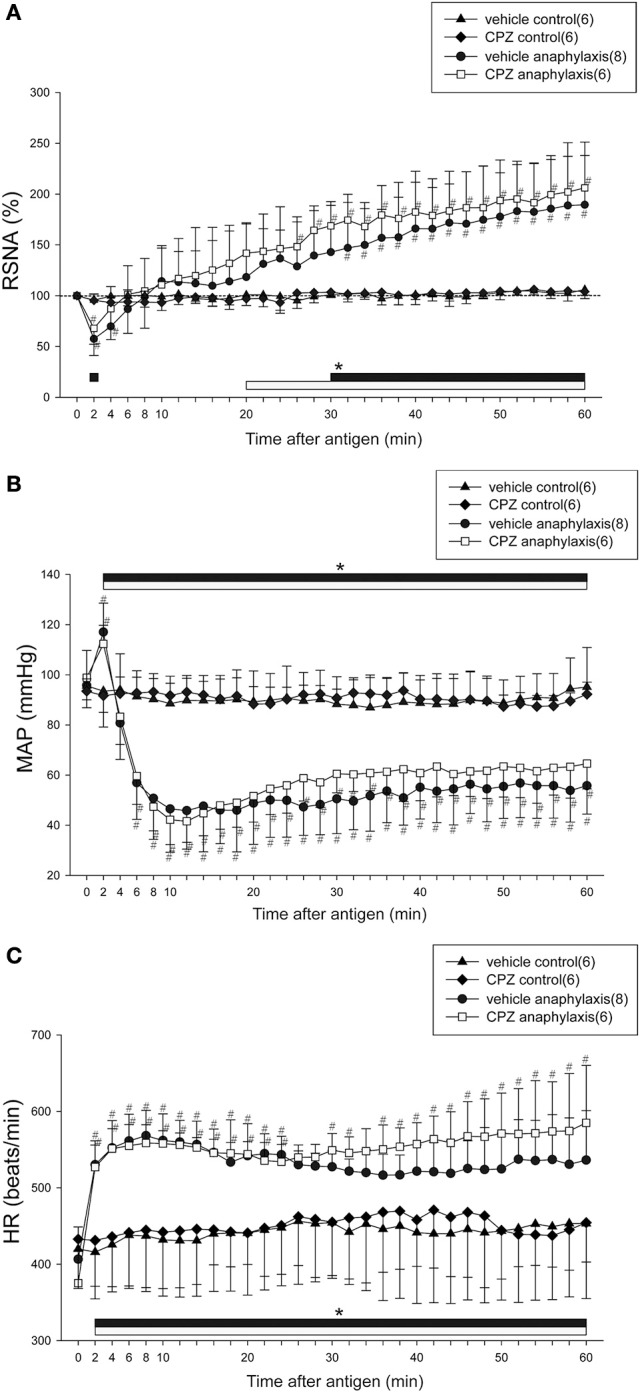
Effects of a TRPV1 blocker on renal sympathetic and cardiovascular responses to anaphylactic hypotension. Time-course data of the changes in RSNA **(A)**, MAP **(B)**, and HR **(C)** after antigen injection in the sensitized and vehicle-pretreated mice (●; vehicle anaphylaxis), the sensitized and CPZ-pretreated mice (□; CPZ anaphylaxis), the nonsensitized and vehicle-pretreated mice (▲; vehicle control), and the nonsensitized and CPZ-pretreated mice (♦; CPZ control). The numbers of mice used are given in parentheses. Values are expressed as means ± SD. ^#^*P* < 0.05 vs. the vehicle control group; ^*^P < 0.05 vs. the baseline; the black bar and white bar show the significant changes in vehicle anaphylaxis group and CPZ anaphylaxis group, respectively.

Figure 3 shows the time-course changes in RSNA, MAP, and HR during anaphylactic shock in mice pretreated with CPZ or vehicle. In the vehicle anaphylaxis group, the biphasic responses of RSNA and MAP to antigen injection, as well as the tachycardia response of HR, were observed similarly to those in the anaphylaxis group, as shown in Figure 1. Pretreatment with CPZ did not affect the renal sympathetic and cardiovascular responses to anaphylaxis; there were no differences in RSNA, MAP, or HR responses between CPZ anaphylaxis group and vehicle anaphylaxis group (Figure 3). The non-sensitized mice pretreated with CPZ or vehicle showed no significant changes in the variables throughout the experimental period (Figure 3).

### Renal sympathetic and cardiovascular responses to anaphylaxis in TRPV1^−/−^ mice

**Figure 4:**
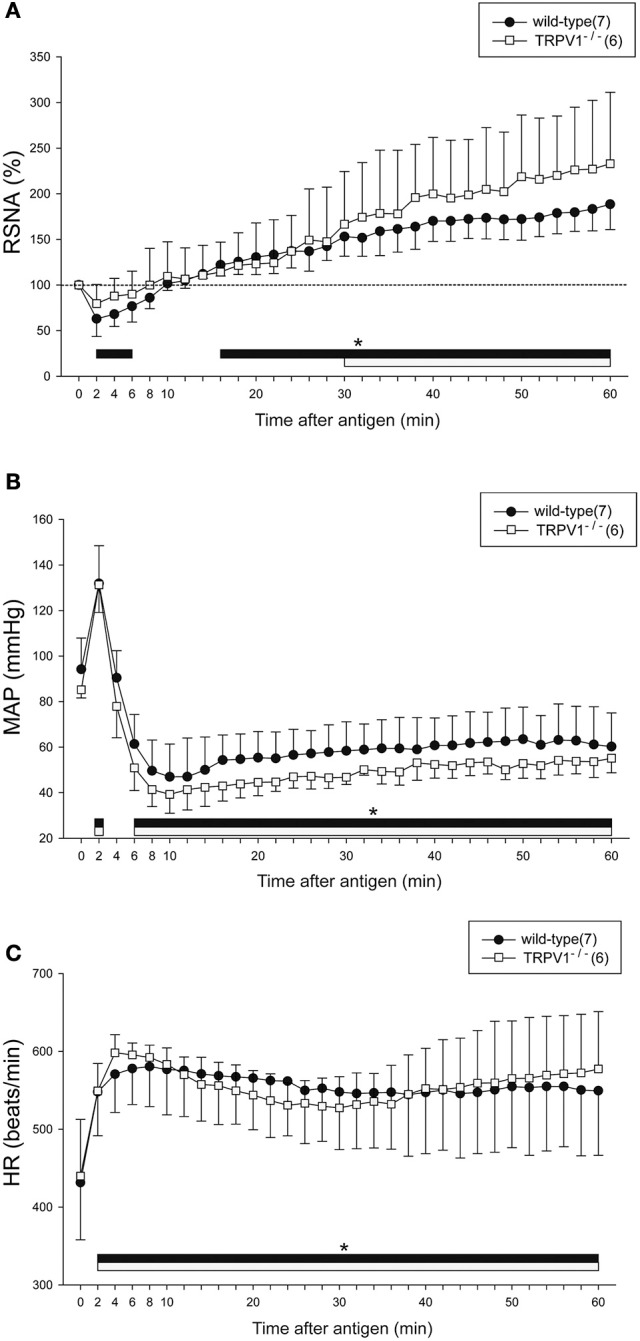
Renal sympathetic and hemodynamic responses to anaphylactic hypotension in TRPV1^−/−^ mice. Time-course data of the changes in RSNA **(A)**, MAP **(B)**, and HR **(C)** during anaphylactic shock in TRPV1−/− mice (□), and wild-type mice (●). The numbers of mice used are given in parentheses. Values are expressed as means ± SD. ^*^P < 0.05 vs. the baseline; the black bar and white bar show the significant changes in wild-type mice and TRPV1^−/−^ mice, respectively.

Figure 4 shows the summarized data of the responses of RSNA, MAP, and HR to anaphylaxis in the wild-type and TRPV1^−/−^ mice. Similar to the responses of the intact mice as shown in Figure 2, the biphasic changes in RSNA and MAP and the sustained increase in HR were observed in wild-type mice. The biphasic response of RSNA to anaphylactic shock was also observed in the TRPV1^−/−^ mice; it began to decrease but not significantly (79 ± 21% of the baseline) within 2 min after antigen injection, and then progressively increased to 230% of the baseline until 60 min (Figure 4A). There were no significant differences in the antigen-induced changes in RSNA, MAP, or HR between the wild-type mice and TRPV1^−/−^ mice (Figure 4).

Regarding baroreflex, MAP changes induced by SNP or PNE caused similar responses of RSNA and HR in the wild-type mice and TRPV1^−/−^mice (SNP-wild-type mice; peak response of RSNA 158 ± 17%, peak response of MAP −50 ± 6 mmHg, peak response of HR 37 ± 16 beats/min, ΔRSNA/ΔMAP = −1.2 ± 0.4, ΔHR/ΔMAP = −0.77 ± 0.33, SNP-TRPV1^−/−^mice; peak response of RSNA 206 ± 37%, peak response of MAP −41 ± 5 mmHg, peak response of HR 41 ± 21 beats/min, ΔRSNA/ΔMAP = −2.66 ± 1.44, ΔHR/ΔMAP = −1.03 ± 0.5, PNE-wild-type mice; peak response of RSNA 63 ± 10%, peak response of MAP 40 ± 6 mmHg, peak response of HR −18 ± 7 beats/min, ΔRSNA/ΔMAP = −0.97 ± 0.4, ΔHR/ΔMAP = −0.45 ± 0.17, PNE-TRPV1^−/−^ mice; peak response of RSNA 67 ± 8%, peak response of MAP 39 ± 7 mmHg, peak response of HR −22 ± 15 beats/min, ΔRSNA/ΔMAP = −0.84 ± 0.23, ΔHR/ΔMAP = −0.56 ± 0.36).

## Discussion

In the present study, we obtained three major findings: (1) the renal sympathetic response to anaphylactic hypotension in anesthetized mice was biphasic, an initial decrease followed by a progressive increase; (2) The initial RSNA inhibition was not affected by either SAD or vagotomy, whereas the subsequent sympathoexcitation was attenuated by SAD, but not by vagotomy; (3) the renal sympathetic and cardiovascular responses to anaphylaxis were preserved both in the mice treated with the TRPV1 antagonist and in the TRPV1^−/−^ mice. These findings suggest that the renal sympathoexcitation at the late stage of anaphylactic hypotension is partly mediated by the afferent pathway of carotid sinus nerve, while either the vagal nerve or TRPV1 is not involved in anaphylaxis-induced changes in RSNA, HR or SAP. To the best of our knowledge, this is the first study to demonstrate the biphasic RSNA response to mouse anaphylactic hypotension and a significant role of the carotid sinus nerves, but not TRPV1 or vagal nerves, in this response.

We have clearly demonstrated that mouse anaphylaxis causes the biphasic RSNA response composed of the initial decrease followed by a progressive increase, and that interestingly, renal sympathetic response to anaphylaxis differs from response to blood pressure manipulation by PNE injection and bleeding. In addition, this response of mice contrasts to those previously reported for rats and dogs: after antigen injection, RSNA monotonically increased in rats (Sun et al., 2014), whereas the RSNA did not increase in dogs (Koyama et al., 1990). Furthermore, the late renal sympathoexcitation was partly baroreflex dependent in mice of the present study, while the similar increase in RSNA of the anesthetized rats was baroreflex independent (Sun et al., 2014). These lines of evidence suggest that there is species difference in renal sympathetic response to anaphylactic hypotension.

It should be noted that SAD did not affect the initial inhibitory RSNA response even in the presence of SAP elevation, suggesting that the initial sympathoinhibition is independent of the baroreceptor reflex. However, this finding is consistent with the previous report that the baroreceptors did not affect the inhibitory RSNA response to anaphylactic hypotension in dogs (Koyama et al., 1990). The reasons why baroreflex was not involved in sympathoinhibitory response at the initial stage of anaphylaxis remain unknown. As a possible explanation, anesthesia attenuates baroreflex reactivity, leading to baroreflex-independent sympathoinhibition. Indeed, we previously reported that RSNA response to SNP-induced hypotension in conscious rats was larger than those in anesthetized rats, and that initial sympathoexcitaion in the presence of rapid SAP fall during anaphylactic hypotension was mediated by the baroreflex in awake rats but not in anesthetized rats (Sun et al., 2014). However, this possibility is unlikely because the baroreceptor reflex was effectively working in the present anesthetized mice as demonstrated by the normal RSNA response to PNE. We here provide more plausible explanations. Nitric oxide (NO) generated in the central nervous system (CNS) during anaphylaxis might inhibit RSNA, independently of the baroreflex system. Consistent with this idea, NO in the hypothalamus elevated during anaphylaxis (Monasterio and Morales, 2011), and a microinjection of an NO agonist, SNP into the hypothalamic nucleus caused suppression of RSNA (Zhang et al., 2001). In addition, inhibition of NO synthase attenuated renal sympathoinhibition during anaphylaxis in dogs (Shibamoto et al., 1996). Thus, increased hypothalamic NO at the initial stage of anaphylaxis might directly inhibit RSNA, overwhelming the baroreflex.

In the present study, although SAD attenuated the renal sympathoexcitation at the late stage of anaphylaxis, RSNA was significantly elevated from 34 min after antigen (Figure 2A), indicating that the late excitatory sympathetic response is only partly due to activation of arterial baroreceptors. This renal sympathoexcitation independent of the baroreceptors may be attributed to the adjustment of CNS. The chemical mediators such as histamine and prostaglandins produced as a result of anaphylactic reactions could increase the sympathetic nerve activity by directly acting on CNS (Tanida et al., 2007; Zhang Z. H. et al., 2011). Moreover, the central chemoreceptors at the brain stem may be activated by severe metabolic acidosis, which was secondary to anaphylactic hypotension, resulting in the delayed excitatory response of RSNA in mice. Taken together, the mechanism for the sympathetic response to anaphylaxis in mice is complex, and may be regulated by both the baroreflex system and CNS. The chemical mediators released during anaphylaxis may have conflicting effects on the central control of sympathetic outflow; further study is required to determine the predominant factors that control the autonomic nervous system at different phases of anaphylaxis in mice.

The SAP response to anaphylaxis in mice is characterized by the initial transient increase, which precedes sustained hypotension (Liu et al., 2007; Wang et al., 2014). We here confirmed this finding in the present study. This initial hypertension is caused by increased cardiac output, but not by vasoconstriction (Wang et al., 2014). The present study also supported this finding: if the initial increase of SAP was produced by peripheral vasoconstriction, the sympathetic outflow to the resistance arteries would be expected to increase. However, RSNA, the sympathetic nerve activity to the renal artery, one of the representative resistance arteries, did not increase but decreased in the present study.

In the present study, although SAD attenuated the renal sympathoexcitation at the late stage in anesthetized mice, it did not exacerbate the anaphylaxis-induced hypotension. Actually, we previously showed that under pentobarbital anesthesia, SAD did not exacerbate anaphylactic hypotension in rats (Sun et al., 2014). Thus, these findings suggest that in anesthetized mice and rats, SAD does not adversely affect anaphylactic hypotension. As a possible explanation, we assume that hypotensive effects of the anaphylactic chemical mediators were too strong to be counteracted by excitation of the sympathetic nervous system at the late stage in mice. Further investigations are required to verify this assumption.

One of the interesting findings in the present study is the dissociation between RSNA inhibition and tachycardia at the early phase of anaphylaxis: HR increased, whereas RSNA decreased immediately after antigen injection. One explanation is that the regional differences may exist between kidney and heart in sympathetic response pattern (Iriki and Simon, 2012). The second possibility is that humoral substances such as anaphylactic chemical mediators (Triggiani et al., 2008) or epinephrine released from the adrenal glands (Zhang W. et al., 2011; Wang et al., 2013) exerted direct positive chronotropic actions on the heart. Finally, the increase in HR at the early stage could be accounted for by the Bainbridge reflex. In the same mouse anaphylaxis models, we observed an increase in the central venous pressure as well as increased cardiac output. Increased venous return might activate the Bainbridge reflex, resulting in tachycardia although the vagotomy did not completely eliminate the increase in HR in the present study (Figure 2C). Of note, the increase in HR and MAP at the initial stage seems to be independent of the effect of the volume intravenously injected, because subcutaneous injections of antigen (10 mg) caused similar responses.

TRPV channels, acting as sensory mediators, play an important role in modulating vascular functions (Baylie and Brayden, 2011). TRPV1 is involved in the regulation of cardiovascular responses to acute hemorrhagic shock in rats via the baroreceptor reflex (Akabori et al., 2007). Afferent neural baroreceptor pathways and the baroreflex signaling may be compromised when TRPV1 is inhibited (Sun et al., 2009). Actually, in the present study, arterial baroreceptors contributed to the increase in RSNA at the late stage of anaphylactic hypotension. Based on these findings, it was expected that TRPV1 participated in the baroreceptor-mediated sympathoexcitation at the late stage of anaphylactic hypotension. However, neither the sympathetic and cardiovascular responses to anaphylaxis nor the baroreflex responses to SNP-induced hypotension were significantly different between the wild-type mice and TRPV1^−/−^ mice. These findings suggest that TRPV1 is not involved in regulation of sympathetic and cardiovascular responses to anaphylaxis in mice.

In conclusion, during anaphylaxis in anesthetized mice, RSNA showed a biphasic response, an initial decrease followed by a sustained increase, while HR a progressive increase. The initial renal sympathoinhibition was independent of carotid sinus baroreceptors, while the sympathoexcitation and tachycardia at the late stage were partly mediated by baroreceptors, but not by vagal nerve or TRPV1. However, anaphylactic hypotension was not affected by carotid sinus nerve, vagal nerve or TRPV1.

## Author contributions

MamT and TS conceived and designed the experiments. MamT and TZ performed all experiments with help by KU, YS, MakT. MamT, TZ, WY, YuK, and YaK discussed the data. MamT, TZ, and TS wrote the manuscript with comment from other authors. All authors approved the final version of the manuscript.

### Conflict of interest statement

The authors declare that the research was conducted in the absence of any commercial or financial relationships that could be construed as a potential conflict of interest.

## Supplementary material

Supplementary Figure 1 Time-course data of the changes in RSNA **(A)**, MAP **(B,C)** and HR **(D)** after subcutaneous injections of antigen in sensitized mice are shown. ●, antigen 0.1 mg; □, antigen 1 mg; ▲, antigen 10 mg. The numbers of mice used are given in parentheses. Values are expressed as means ± SD. ^#^*P* < 0.05 vs. the antigen 0.1 mg group. ^*^*P* < 0.05 vs. the baseline; the white bar and gray bar show the significant changes in antigen 1 mg group and antigen 10 mg group, respectively.

## References

[B1] Akabori H. Yamamoto H. Tsuchihashi H. Mori T. Fujino K. Shimizu T.. (2007). .Transient receptor potential vanilloid 1 antagonist, capsazepine, improves survival in a rat hemorrhagic shock model. Ann. Surg. 245, 964–970. 10.1097/01.sla.0000255577.80800.e1 17522523 PMC1876964

[B2] Baylie R. L. Brayden J. E. (2011). TRPV channels and vascular function. Acta Physiol. (Oxf). 203, 99–116. 10.1111/j.1748-1716.2010.02217.x 21062421 PMC3134601

[B3] Brown A. F. (1995). Anaphylactic shock: mechanisms and treatment. J. Accid. Emerg. Med. 12, 89–100. 10.1136/emj.12.2.89 7582425 PMC1342543

[B4] Castex N. Fioramonti J. Fargeas M. J. Bueno L. (1995). c-fos expression in specific rat brain nuclei after intestinal anaphylaxis: involvement of 5-HT3 receptors and vagal afferent fibers. Brain Res. 688, 149–160. 10.1016/0006-8993(95)00526-V 8542301

[B5] Caterina M. J. Schumacher M. A. Tominaga M. Rosen T. A. Levine J. D. Julius D. (1997). The capsaicin receptor: a heat-activated ion channel in the pain pathway. Nature 389, 816–824. 10.1038/39807 9349813

[B6] Cauwels A. Janssen B. Buys E. Sips P. Brouckaert P. (2006). Anaphylactic shock depends on PI3K and eNOS-derived NO. J. Clin. Invest. 116, 2244–2251. 10.1172/JCI25426 16886062 PMC1523420

[B7] Cui H. Okamoto Y. Yoshioka K. Du W. Takuwa N. Zhang W.. (2013). Sphingosine-1-phosphate receptor 2 protects against anaphylactic shock through suppression of endothelial nitric oxide synthase in mice. J. Allergy Clin. Immunol. 132, 1205–1214. 10.1016/j.jaci.2013.07.026 24021572

[B8] Hermes S. M. Andresen M. C. Aicher S. A. (2016). Localization of TRPV1 and P2X3 in unmyelinated and myelinated vagal afferents in the rat. J. Chem. Neuroanat. 72, 1–7. 10.1016/j.jchemneu.2015.12.003 26706222 PMC4764453

[B9] Iriki M. Simon E. (2012). Differential control of efferent sympathetic activity revisited. J. Physiol. Sci. 62, 275–298. 10.1007/s12576-012-0208-9 22614392 PMC10717676

[B10] Jacobsen J. Johnsen C. R. Skov P. S. Warberg J. Knigge U. Secher N. H. (1995). Cardiovascular and hormonal responses to anaphylactic shock in the pig. Clin. Physiol. 15, 81–90. 10.1111/j.1475-097X.1995.tb00432.x 7712695

[B11] Kim B. M. Lee S. H. Shim W. S. Oh U. (2004). Histamine-induced Ca2+ influx via the PLA2/lipoxygenase/TRPV1 pathway in rat sensory neurons. Neurosci. Lett. 361, 159–162. 10.1016/j.neulet.2004.01.019 15135918

[B12] Koyama S. Fujita T. Uematsu H. Shibamoto T. Aibiki M. Kojima S. (1990). Inhibitory effect of renal nerve activity during canine anaphylactic hypotension. *Am. J*. Physiol. 258, R383–R387. 10.1152/ajpregu.1990.258.2.R383 2309932

[B13] Liu W. Takano H. Shibamoto T. Cui S. Zhao Z. S. Zhang W.. (2007). Involvement of splanchnic vascular bed in anaphylactic hypotension in anesthetized BALB/c mice. Am. J. Physiol. Regul. Integr. Comp. Physiol. 293, R1947–R1953. 10.1152/ajpregu.00904.2006 17715178

[B14] Marotta D. M. Costa R. Motta E. M. Fernandes E. S. Medeiros R. Quintão N. L.. (2009). Mechanisms underlying the nociceptive responses induced by platelet-activating factor (PAF) in the rat paw. Biochem. Pharmacol. 77, 1223–1235. 10.1016/j.bcp.2008.12.025 19283893

[B15] Monasterio N. Morales T. (2011). Nitric oxide has a role in attenuating the neuroendocrine response to anaphylactoid stress during lactation. Brain Res. 1402, 54–66. 10.1016/j.brainres.2011.05.062 21696708

[B16] Moriyama T. Higashi T. Togashi K. Iida T. Segi E. Sugimoto Y.. (2005). Sensitization of TRPV1 by EP1 and IP reveals peripheral nociceptive mechanism of prostaglandins. Mol. Pain. 1:3. 10.1186/1744-8069-1-3 15813989 PMC1074353

[B17] Numazaki M. Tominaga M. (2004). Nociception and TRP Channels. Curr. Drug Targets CNS. Neurol. Disord. 3, 479–485. 10.2174/1568007043336789 15578965

[B18] Potas J. R. Briscoe H. Horiuchi J. Killinger S. Dampney R. A. (2004). Renal sympathetic and cardiac changes associated with anaphylactic hypotension. Auton. Neurosci. 112, 25–30. 10.1016/j.autneu.2004.03.004 15233927

[B19] Salzer I. Gantumur E. Yousuf A. Boehm S. (2016). Control of sensory neuron excitability by serotonin involves 5HT2C receptors and Ca2+-activated chloride channels. Neuropharmacology. 110, 277–286. 10.1016/j.neuropharm.2016.08.006 27511837 PMC6192515

[B20] Shibamoto T. Wang H. G. Tanaka S. Miyahara T. Koyama S. (1996). Participation of nitric oxide in the sympathetic response to anaphylactic hypotension in anesthetized dogs. Neurosci. Lett. 212, 99–102. 10.1016/0304-3940(96)12782-8 8832648

[B21] Shim W. S. Tak M. H. Lee M. H. Kim M. Kim M. Koo J. Y.. (2007). TRPV1 mediates histamine-induced itching via the activation of phospholipase A2 and 12-lipoxygenase. J. Neurosci. 27, 2331–2337. 10.1523/JNEUROSCI.4643-06.2007 17329430 PMC6673467

[B22] Smith P. K. Nilius B. (2013). Transient receptor potentials (TRPs) and anaphylaxis. Curr. Allergy Asthma Rep. 13, 93–100. 10.1007/s11882-012-0301-4 22972391

[B23] Song J. Tanida M. Shibamoto T. Zhang T. Wang M. Kuda Y.. (2016). The role of lumbar sympathetic nerves in regulation of blood flow to skeletal muscle during anaphylactic hypotension in anesthetized rats. PLoS ONE 11:e0150882. 10.1371/journal.pone.0150882. 26998924 PMC4801202

[B24] Sun H. Li D. P. Chen S. R. Hittelman W. N. Pan H. L. (2009). Sensing of blood pressure increase by transient receptor potential vanilloid 1 receptors on baroreceptors. J. Pharmacol. Exp. Ther. 331, 851–859. 10.1124/jpet.109.160473 19726694 PMC2784714

[B25] Sun L. Tanida M. Wang M. Kuda Y. Kurata Y. Shibamoto T. (2014). Effects of anesthetics on the renal sympathetic response to anaphylactic hypotension in rats. PLoS ONE 9:e113945. 10.1371/journal.pone.0113945 25423366 PMC4244183

[B26] Tanida M. Hayata A. Shintani N. Yamamoto N. Kurata Y. Shibamoto T.. (2013). Central PACAP mediates the sympathetic effects of leptin in a tissue-specific manner. Neuroscience 238, 297–304. 10.1016/j.neuroscience.2013.02.016 23454538 PMC5684871

[B27] Tanida M. Kaneko H. Shen J. Nagai K. (2007). Involvement of the histaminergic system in renal sympathetic and cardiovascular responses to leptin and ghrelin. Neurosci. Lett. 413, 88–92. 10.1016/j.neulet.2006.11.035 17166664

[B28] Tominaga M. Caterina M. J. Malmberg A. B. Rosen T. A. Gilbert H. Skinner K.. (1998). The cloned capsaicin receptor integrates multiple pain-producing stimuli. Neuron 21, 531–543. 10.1016/S0896-6273(00)80564-4 9768840

[B29] Triggiani M. Patella V. Staiano R. I. Granata F. Marone G. (2008). Allergy and the cardiovascular system. Clin. Exp. Immunol. 153, 7–11. 10.1111/j.1365-2249.2008.03714.x 18721322 PMC2515352

[B30] Wang M. Shibamoto T. Tanida M. Kuda Y. Kurata Y. (2014). Mouse anaphylactic shock is caused by reduced cardiac output, but not by systemic vasodilatation or pulmonary vasoconstriction, via PAF and histamine. Life Sci. 116, 98–105. 10.1016/j.lfs.2014.09.010 25252221

[B31] Wang M. Tanida M. Shibamoto T. Kurata Y. (2013). Alpha-adrenoceptor antagonists and chemical sympathectomy exacerbate anaphylaxis-induced hypotension, but not portal hypertension, in anesthetized rats. Am. J. Physiol. Regul. Integr. Comp. Physiol. 305, R900–R907. 10.1152/ajpregu.00120.2013 23948775

[B32] Wang Y. Novotny M. Quaiserová-Mocko V. Swain G. M. Wang D. H. (2008). TRPV1-mediated protection against endotoxin-induced hypotension and mortality in rats. Am. J. Physiol. Regul. Integr. Comp. Physiol. 294, R1517–R1523. 10.1152/ajpregu.00005.2008 18337316 PMC2668825

[B33] Zhang K. Li Y. F. Patel K. P. (2001). Blunted nitric oxide-mediated inhibition of renal nerve discharge within PVN of rats with heart failure. *Am. J. Physiol. Heart Circ*. Physiol. 281, H995–H1004. 10.1152/ajpheart.2001.281.3.H995 11514264

[B34] Zhang L. Jones S. Brody K. Costa M. Brookes S. J. (2004). Thermosensitive transient receptor potential channels in vagal afferent neurons of the mouse. Am. J. Physiol. Gastrointest. Liver Physiol. 286, G983–G991. 10.1152/ajpgi.00441.2003 14726308

[B35] Zhang T. Tanida M. Uchida K. Suzuki Y. Yang W. Kuda Y.. (2017). Biphasic renal sympathetic response to hemorrhagic hypotension in mice. Shock. [Epub ahead of print]. 10.1097/SHK.0000000000000889 28459715

[B36] Zhang W. Shibamoto T. Kuda Y. Ohmukai C. Kurata Y. (2011). Pulmonary vasoconstrictive and bronchoconstrictive responses to anaphylaxis are weakened via β2-adrenoceptor activation by endogenous epinephrine in anesthetized rats. Anesthesiology. 114, 614–623. 10.1097/ALN.0b013e31820b8d34 21307766

[B37] Zhang Z. H. Yu Y. Wei S. G. Nakamura Y. Nakamura K. Felder R. B. (2011). EP_3_ receptors mediate PGE_2_-induced hypothalamic paraventricular nucleus excitation and sympathetic activation. Am. J. Physiol. Heart Circ. Physiol. 301, H1559–H1569. 10.1152/ajpheart.00262.2011 21803943 PMC3197370

