# Supplementary figures and images for: Mouse Anaphylactic Hypotension Is Characterized by Initial Baroreflex Independent Renal Sympathoinhibition Followed by Sustained Renal Sympathoexcitation
Source: Front Physiol. 2017 Sep 7;8:669. doi: 10.3389/fphys.2017.00669 (PMC5594092; doi:10.3389/fphys.2017.00669)

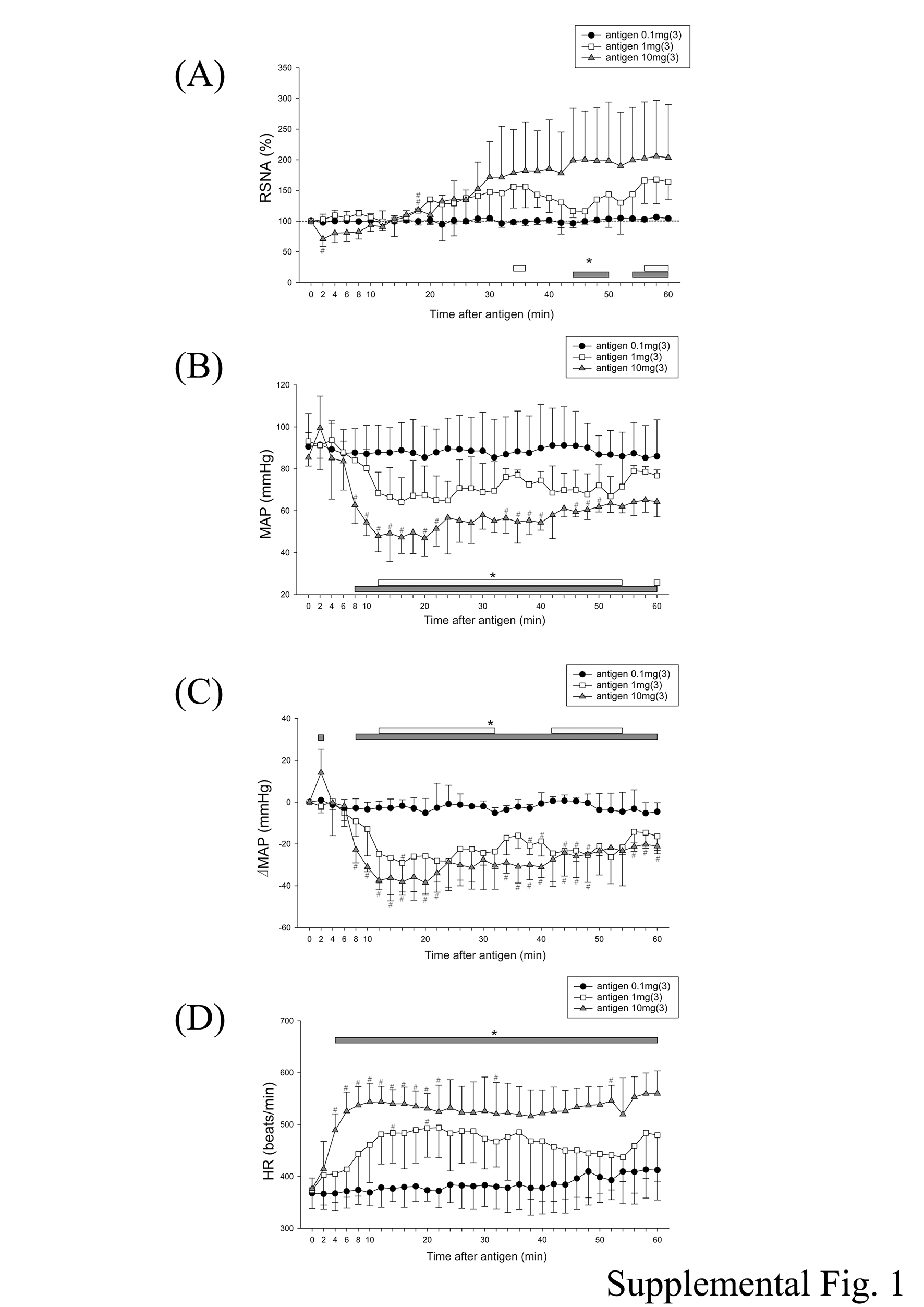

Supplement: Supplementary Figure 1 — Time-course data of the changes in RSNA (A), MAP (B,C) and HR (D) after subcutaneous injections of antigen in sensitized mice are shown. ●, antigen 0.1 mg; □, antigen 1 mg; ▲, antigen 10 mg. The numbers of mice used are given in parentheses. Values are expressed as means ± SD. #P < 0.05 vs. the antigen 0.1 mg group. *P < 0.05 vs. the baseline; the white bar and gray bar show the significant changes in antigen 1 mg group and antigen 10 mg group, respectively. [file Image1.TIF]
